# Andrographolide attenuates skeletal muscle dystrophy in *mdx* mice and increases efficiency of cell therapy by reducing fibrosis

**DOI:** 10.1186/2044-5040-4-6

**Published:** 2014-03-21

**Authors:** Daniel Cabrera, Jaime Gutiérrez, Claudio Cabello-Verrugio, Maria Gabriela Morales, Sergio Mezzano, Ricardo Fadic, Juan Carlos Casar, Juan L Hancke, Enrique Brandan

**Affiliations:** 1 Centro de Regulación Celular y Patología (CRCP), Centro de Regeneración y Envejecimiento (CARE), Laboratorio de Diferenciación Celular y Patología, Departamento de Biología Celular y Molecular, MIFAB, Pontificia Universidad Católica de Chile, Avenida Libertador Bernardo O’Higgins, 340, Santiago, Chile; 2 Departamento de Ciencias Químicas y Biológicas, Universidad Bernardo O’Higgins, Santiago, Chile; 3 Laboratorio de Biología y Fisiopatología Molecular, Departamento de Ciencias Biológicas, Facultad de Ciencias Biológicas & Facultad de Medicina, Universidad Andrés Bello, Santiago, Chile; 4 División de Nefrología, Escuela de Medicina, Universidad Austral, Valdivia, Chile; 5 Departamento de Neurología, Facultad de Medicina, Pontificia Universidad Católica de Chile, Santiago, Chile; 6 Instituto de Farmacología, Universidad Austral de Chile, Valdivia, Chile

**Keywords:** Andrographolide, *mdx*, DMD, Fibrosis, Skeletal muscle, Cell therapy

## Abstract

**Background:**

Duchenne muscular dystrophy (DMD) is characterized by the absence of the cytoskeletal protein dystrophin, muscle wasting, increased transforming growth factor type beta (TGF-β) signaling, and fibrosis. At the present time, the only clinically validated treatments for DMD are glucocorticoids. These drugs prolong muscle strength and ambulation of patients for a short term only and have severe adverse effects. Andrographolide, a bicyclic diterpenoid lactone, has traditionally been used for the treatment of colds, fever, laryngitis, and other infections with no or minimal side effects. We determined whether andrographolide treatment of *mdx* mice, an animal model for DMD, affects muscle damage, physiology, fibrosis, and efficiency of cell therapy.

**Methods:**

*mdx* mice were treated with andrographolide for three months and skeletal muscle histology, creatine kinase activity, and permeability of muscle fibers were evaluated. Fibrosis and TGF-β signaling were evaluated by indirect immunofluorescence and Western blot analyses. Muscle strength was determined in isolated skeletal muscles and by a running test. Efficiency of cell therapy was determined by grafting isolated skeletal muscle satellite cells onto the tibialis anterior of *mdx* mice.

**Results:**

*mdx* mice treated with andrographolide exhibited less severe muscular dystrophy than untreated dystrophic mice. They performed better in an exercise endurance test and had improved muscle strength in isolated muscles, reduced skeletal muscle impairment, diminished fibrosis and a significant reduction in TGF-β signaling. Moreover, andrographolide treatment of *mdx* mice improved grafting efficiency upon intramuscular injection of dystrophin-positive satellite cells.

**Conclusions:**

These results suggest that andrographolide could be used to improve quality of life in individuals with DMD.

## Background

Muscular dystrophies are a group of genetic muscular diseases. The most severe is Duchenne muscular dystrophy (DMD), an X-linked recessive disorder affecting 1 in 3,500 births for which there is no effective therapy [1]. DMD is caused by the absence of dystrophin, a cytoskeletal protein that anchors the muscle fiber to the extracellular matrix (ECM). The absence of this protein increases susceptibility to muscle fiber rupture caused by the continuous cycles of contraction and relaxation [2,3]. Thus, children with this condition gradually and progressively lose muscle strength, typically requiring the use of a wheel chair from the age of ten and dying in the late second or early third decade of life as a result of cardiorespiratory arrest. One of the causes of muscle damage and loss of function is the development of fibrosis, which is characterized by excessive accumulation of extracellular matrix (ECM) that replaces muscle tissue with connective tissue, dramatically affecting the environment of the fibers and normal muscle physiology [4-9].

Pathologic features of DMD include myofiber atrophy, fatty degeneration, necrosis and fibrosis, but only fibrosis has been shown through clinical studies to correlate with poor motor outcome, evaluated by muscle strength and age at loss of ambulation [10]. This finding supports the notion that fibrosis directly contributes to progressive muscle dysfunction and the lethal phenotype of DMD [6]. Therefore, the development of new drugs and therapies with anti-fibrotic activity is crucial.

Andrographolide, a bicyclic diterpenoid lactone, is the major constituent of *Andrographis paniculata*, a plant indigenous to Southeast Asian countries that has been used as an official herbal medicine in China for many years [11]. Traditionally, it is used for the treatment of colds, fever, laryngitis, and other infections, with no or minimal side effects. It has been reported to be particularly efficient at regulating immune responses [12,13] and possesses anti-inflammatory properties by reducing the generation of reactive oxygen species in human neutrophils [11]. Andrographolide not only regulates inflammation, but is also effective against the fibrotic pathology observed in chronic liver and kidney diseases [14-16]. Mechanistically, andrographolide forms a covalent adduct with NF-κB, thus blocking the binding of NF-κB to nuclear proteins [17]. NF-κB is an important transcription factor in the progression of skeletal muscular dystrophic diseases [18-20].

Because dystrophic disorders such as DMD have genetic origins there is a great effort to restore gene expression through gene and/or cell therapies. Nevertheless, these therapies represent a major challenge because muscle is the most abundant tissue in the body. Moreover, fibrosis strongly reduces the efficacy of these approaches [6,9,21], therefore, even if current trials are successful, they are unlikely to elicit a significant benefit when extended to people with more advanced stages of the disease and enhanced fibrosis [21,22]. Therefore, understanding the cellular and molecular mechanisms underlying muscle fibrogenesis associated with muscular dystrophies is critical to the development of an effective anti-fibrotic therapy for this type of disease.

In this study, we investigated the effects of andrographolide on the onset of dystrophy *in mdx* mice, an animal model used to study DMD. We demonstrated that andrographolide treatment of dystrophic mice prevented damage and fibrosis progression as reflected by reduced collagen and fibronectin deposition through a mechanism that involves a decrease in the expression of transforming growth factor type beta (TGF-β) and its downstream mediator connective tissue growth factor (CTGF/CCN2). The reduction of fibrosis was associated with enhanced muscle strength and improved exercise performance. Finally, we determined that the use of andrographolide increased the efficiency of cell therapy through fibrosis reduction.

## Methods

### Animals and experimental exercise

We used 12-week-old control or *mdx* male mice of the C57BL/10 ScSn strain. The animals were kept at room temperature with a 24-hour night-day cycle and were fed with pellets and water *ad libitum*. Experimental exercise involved running the mice on a treadmill three times per week for 30 minutes each session at 12 m/minute over three or four months [6,23-25]. Two experimental groups were designed: animals in the first group were injected intraperitoneally (ip) with andrographolide (1.0 mg/kg/day) and animals in the second group were treated with vehicle alone. At the end of the experiment the muscles were dissected and removed under anesthesia (isofluorane gas) and then the animals were sacrificed. Tissues were used for electrophysiological measurement or rapidly frozen and stored at −80°C until processing. All protocols were conducted in strict accordance with guidelines and with the formal approval of the Animal Ethics Committee of the Pontificia Universidad Católica de Chile.

### Skeletal muscle histology

Muscle architecture and histology were analyzed by H&E staining of transverse sections of muscle [6,26-28].

### Evans blue dye (EBD) uptake

Animals were sacrificed 24 hours after injection with EBD (1% in PBS). The tibialis anterior (TA) muscles were snap-frozen in isopentane, sectioned in 7-μm cryosections, and fixed in 4% paraformaldehyde [6]. Muscle cross-sections were visualized under a Nikon Diaphot Eclipse-600 (Nikon, Melville, NY, USA) inverted microscope equipped for epifluorescence. The percentage of EBD-positive fibers was manually counted in a blinded manner [29].

### Serum creatine kinase measurement

Mice were anesthetized and blood was obtained from the periorbital vascular plexus directly into 70 μl microhematocrit tubes (Fisher Scientific, Loughborough, UK). Serum was obtained by allowing the blood to clot at room temperature for 30 minutes followed by centrifugation at 1,700 × g for ten minutes. Serum creatine kinase (CK) was measured using an enzymatic system (Valtek, Santiago, Chile) according to the manufacturer’s instructions [6].

### Immunoblot analysis

Muscles were homogenized in ten volumes of Tris-EDTA buffer with 1 mM PMSF as described previously [26]. Briefly, protein concentration in aliquots of muscle extract was determined using the bicinchoninic acid protein assay kit (Pierce, Rockford, IL, USA) using BSA as a standard. Aliquots (50 to 100 μg) were subjected to SDS gel electrophoresis in 8% or 10% polyacrylamide gels, electrophoretically transferred onto PVDF membranes (Schleicher & Schuell, Keene, NH, USA), and probed with specific antibodies against fibronectin (Sigma, St. Louis, MO, USA), collagen III (Rockland, Gilbertsville, PA USA) and GAPDH (Millipore, Billerica, MA, USA), tubulin (Sigma, St. Louis, MO, USA) as described previously [6]. All immunoreactions were visualized using an enhanced chemiluminescence kit (Pierce, Rockford, IL, USA). Densitometric analysis and quantification were performed using ImageJ software (NIH, Bethesda, MD, USA) [25].

### Immunofluorescence microscopy

For immunofluorescence, 7-μm cryosections were fixed in 4% paraformaldehyde, blocked for one hour in 10% goat serum in PBS and incubated for one hour at room temperature with specific antibodies against fibronectin (Sigma, St. Louis, MO, USA), collagen I (Chemicon, Temecula, CA, USA), F4/80 (Abcam, Cambridge, MA, USA), p-Smad-2 (Abcam, Cambridge, MA, USA), and dystrophin (Santa Cruz Biotechnology, Santa Cruz, CA, USA). FITC-conjugated goat anti-rabbit IgG and rabbit anti-mouse IgG (Invitrogen, Carlsbad, CA, USA) were used as secondary antibodies. For monoclonal anti-mouse antibodies, all incubations were performed with mouse IgG-blocking solution from the MOM kit (Vector Lab, Burlingame, CA, USA) diluted in 0.01% Triton X-100/PBS. For nuclear staining, sections were incubated with 1 μg/ml Hoechst 33258 in PBS for ten minutes. After rinsing, the coverslips were mounted using Fluoromount (Dako, Carpinteria, CA, USA) and observed under a Nikon Diaphot inverted microscope equipped for epifluorescence [26].

### NF-κB detection *in vivo* by Southwestern blotting

Synthetic sense DNA 5′-AGTTGAGGGGACTTTCCCAGGC-3′, which contains a consensus sequence for NF-κB, was used as the probe. After annealing with complementary DNA (80°C for two minutes), the probe was labeled with digoxigenin (DIG) oligonucleotide 3′-end labeling (Boehringer Mannheim, Mannheim, Germany). Paraffin-embedded muscle sections were dewaxed, rehydrated, and fixed with 0.2% paraformaldehyde for 30 minutes at 28°C. Sections were subsequently digested with 433 U/mg pepsin A (Sigma, St. Louis, MO, USA), washed twice with buffer 1 (10 mmol/L HEPES, 40 mmol/L NaCl, 10 mmol/L MgCl_2_, 1 mmol/L DTT, 1 mmol/L EDTA, 0.25% BSA, pH 7.4), treated with 0.1 mg/mL DNase I and washed with buffer 2 (10 mmol/L HEPES, 40 mmol/L NaCl, 1 mmol/L DTT, 10 mmol/L EDTA, 0.25% BSA, pH 7.4) to stop the reaction. Labeled probe (100 pmol/L) diluted in buffer 1 containing 0.5 mg/mL poly dl-dC (Pharmacia, New York, NY, USA) was applied overnight at 37°C. After washing, sections were incubated for one hour in blocking solution (0.01× SSC, 0.01% SDS, 0.03% Tween 20, 0.1 mol/L maleic acid, 0.15 mol/L NaCl, pH 7.5) and then overnight at 4°C with rabbit anti-digoxigenin antibody (1:250 in blocking solution; Invitrogen, Carlsbad, CA, USA). The samples were washed and incubated with a secondary Alexa fluor 568 anti-rabbit antibody (Invitrogen, Carlsbad, CA, USA). Nuclear staining with Hoechst 33258 was performed as described above. We used the following negative controls: (a) absence of probes, (b) DIG-labeled mutant NF-κB probe (sense: 5′-AGTTGAGGCTCCTTTCCCAGGC-3′), at the same concentration as labeled probe, (c) competition assays with 100-fold excess of unlabeled NF-κB probe, followed by incubation with the respective labeled probe [30].

### RNA isolation and quantitative real-time PCR analysis

During tissue collection, one TA muscle from each animal was rapidly frozen in liquid nitrogen and used for RNA isolation. Total RNA was isolated with TRIzol reagent according to the manufacturer’s protocol (Invitrogen, Carlsbad, CA, USA). Total RNA (500 ng) from each sample was reverse transcribed to cDNA using Super Script Reverse Transcriptase II (Invitrogen, Carlsbad, CA, USA). Quantitative real-time PCR (qPCR) reactions were performed using SYBR Green Master Mix (Bio-Rad, Hercules, CA, USA). Levels of TGF-β and CTGF were determined as described before [31]. The real-time amplification of genes was measured with the iCycler thermocycler system and iQ5 optical system software (Bio-Rad, Hercules, CA, USA) [32].

### Contractile properties

The isometric force of isolated muscles was measured as described previously [6,25,26]. Briefly, optimum muscle length (Lo) and stimulation voltage were determined from micromanipulation of muscle length to produce maximum isometric twitch force. Maximum isometric tetanic force (Po) was determined from the plateau of the frequency-force relationship after successive stimulations at 1 to 200 Hz for 450 ms, with two-minute rests between stimuli. After determination of isometric contractile properties, muscles were subjected to a three repeated tetanic stimulation protocol. Muscles at Lo were maximally stimulated for 450 ms once every five seconds. After functional testing, muscles were removed from the bath, trimmed of their tendons and any adhering non-muscle tissue, blotted once on filter paper and weighed [33-35]. Muscle mass and Lo were used to calculate specific net force (force normalized per total muscle fiber cross-sectional area (CSA), mN/mm^2^) [6,26].

### Running test

Mice were subjected to a running test for 15 minutes at 15 m/minute on a treadmill. The number of times that mice were retarded to the first one third of the moving platform (detentions) was counted [6,25].

### Single myofiber isolation and satellite cell grafting

Single myofibers were isolated essentially as described previously [6,36]. Briefly, extensor digitorum longus (EDL) and soleus muscles from six-week-old C57-BL10 mice were dissected and digested in 0.2% (w/v) collagenase type 1 (Sigma, St. Louis, MO, USA) in DMEM (Gibco, Grand Island, NY, USA) with 4 mM L-glutamine (Sigma, St. Louis, MO, USA) and 1% penicillin and streptomycin solution (Sigma, St. Louis, MO, USA) for 90 minutes in a 37°C water bath. Satellite cells were separated from the myofibers by physical trituration using the method of Collins *et al*. [36]. The isolated intact fibers were suspended in 10 ml of complete medium and triturated with a 19 G needle mounted on a 1 ml syringe. The suspension was sequentially passed through a 70-μm and 40-μm cell sieve (BD Biosciences, San Jose, CA, USA) to remove debris. The satellite cell suspension was centrifuged for 15 minutes at 450 × g. The pellet was resuspended in physiologic serum (0.9% NaCl). An aliquot was stained with 1 μg/ml Hoechst and 1 μg/ml cholera toxin subunit B conjugated to Alexa Fluor 488 (Invitrogen, Carlsbad, CA, USA) for five minutes, washed with PBS and incubated with trypan blue. Cells were counted using a hemocytometer. Double-stained cells that exclude trypan blue were counted as viable cells and the concentration of cells was adjusted to 25 cells/μl. To confirm the purity of the isolated satellite cells, an aliquot was seeded onto Matrigel (1 mg/ml) (Sigma, St. Louis, MO, USA) and cultured overnight in complete medium for 18 hours before immunocytochemistry for myogenic markers. For grafting, 500 satellite cells were grafted into both TA muscles of seven-month-old *mdx* mice in a C57-BL10 background under anesthesia using an 8-mm 30 G needle under microscopic observation.The number of dystrophin positive fibers was determined as described previously [6].

### Determination of the grafted-satellite cell survival

The determination of the survival of the grafted satellite cells was performed as previously described [6]. Briefly, 500 freshly isolated satellite cells from a C57-EGFP mice, as described in the previous section, were grafted into both TA muscles of seven-month-old *mdx* mice in a C57-BL10 background under anesthesia using an 8-mm 30 G needle under microscopic observation. The muscle genomic DNA was extracted with a DNA purification kit (QiAamp DNA from Quiagen). Then the purified DNA were subjected to amplification by real time PCR using TaqMan PCR Universal Master Mix, in an Illumina Eco real time PCR (Illumina, USA), Primer and Taqman probe for EGFP and b-actin (endogenous control) were from Applied Biosystems, (USA).

### Statistics

The statistical significance of differences between the means of the experimental groups was evaluated using one-way analysis of variance (ANOVA) with a *post hoc* Bonferroni multiple-comparison test or two tailed *t*-tests (Prism 3.0, GraphPad, San Diego, CA, USA). A difference was considered statistically significant at *P* < 0.05.

## Results

### Andrographolide treatment improves histology and reduces muscle damage in dystrophic skeletal muscle

**Figure 1:**
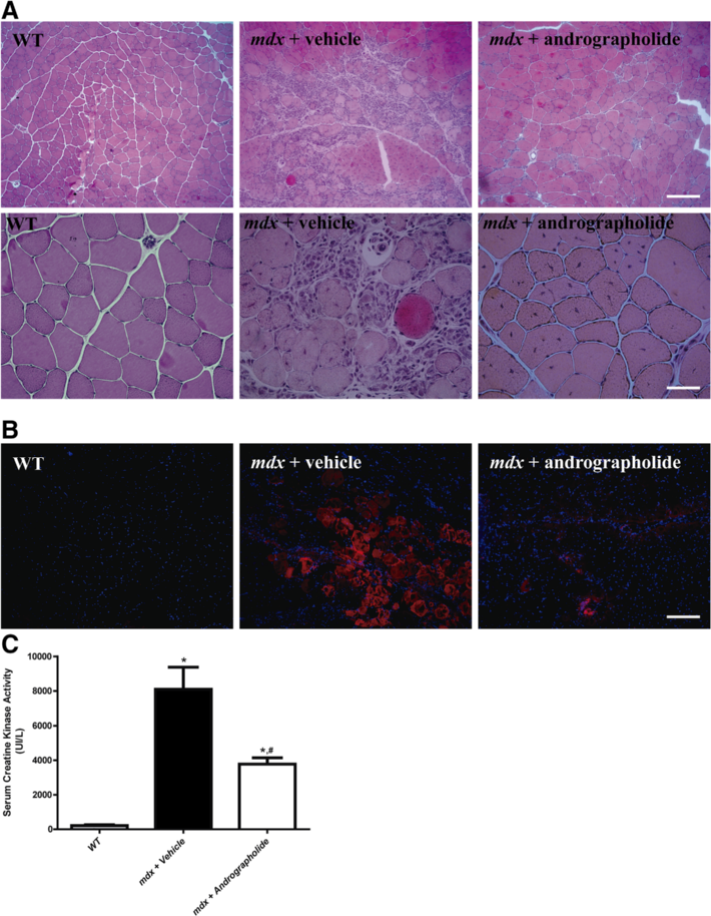
**Andrographolide reduces skeletal muscle damage in *mdx *mice.** To augment the extent of muscle fibrosis, three-month-old *mdx* mice were subjected to an exercise protocol for three months [6,25]. During this period, mice were treated with 1 mg/kg andrographolide or vehicle (intraperitoneal (ip) injections three times per week, six animals per group). **(A)** H&E staining of tibialis anterior (TA) muscles showed a striking reduction in the damaged areas of muscle in andrographolide-treated *mdx* mice compared with untreated *mdx* mice. Upper panel shows 100X magnification pictures (scale bars = 200 μm), while the bottom panel shows 400X magnification pictures (scale bars = 50 μm). **(B)** Evans blue dye uptake in TA muscle fibers from wild type (WT), vehicle-treated *mdx* mice, and andrographolide-treated *mdx* mice. Nuclei were labeled with Hoechst. Mice were injected ip with 1% Evans blue dye 24 hours before muscle fixation (scale bar = 200 μm). **(C)** Serum creatine kinase (CK) was measured to evaluate skeletal muscle damage. The bar graph shows a significant reduction in serum CK activity in andrographolide-treated *mdx* mice compared with vehicle-treated *mdx* mice. Values are expressed as mean ± SD of three independent experiments, using five mice for each experimental condition. (*P < 0.05 relative to WT mice; #*P* < 0.05 relative to vehicle-treated *mdx* mice). The recovery score was 49.5%.

To determine whether andrographolide has an effect on the dystrophic phenotype of *mdx* mice, we evaluated the histology of the TA muscle from andrographolide and vehicle-treated *mdx* mice by H&E staining. Administration of andrographolide to *mdx* mice for three months reduced the increase in infiltrating cells and necrotic areas and showed a clear improvement in muscle histopathology compared with vehicle-treated *mdx* mice (Figure 1A, two different magnifications). To specifically evaluate damage at the *mdx* sarcolemma, we used the EBD uptake protocol [29]. Lower EBD uptake was observed in the muscle fibers from *mdx* mice treated with andrographolide compared with control *mdx* fibers, suggesting less muscle damage (Figure 1B). Concordantly, serum CK levels were decreased in andrographolide-treated *mdx* compared with control *mdx* mice, with an approximately 50% recovery score [37] (Figure 1C). Table 1 shows quantification of areas of necrosis and regeneration [38]. Administration of andrographolide to *mdx* decreased cumulative damage. We did not find any differences in the minimal Feret’s diameter between *mdx* mice treated with andrographolide and *mdx* mice treated with vehicle (Additional file 1: Figure S1). These results indicate that treatment of *mdx* mice with andrographolide improves the architecture of dystrophic skeletal muscles and decreases tissue damage.

**Table 1:** **Cumulative muscle damage in exercised *mdx *** **mice treated or not with andrographolide**

	**Necrosis**	**Regeneration**	**Cumulative damage**
*mdx* + vehicle	7.63 ± 0.85	42.14 ± 4.03	49.77 ± 4.61
*mdx* + andrographolide	4.14 ± 0.51^b^	30.23 ± 2.86^a^	34.37 ± 2.97^a^

Cumulative skeletal muscle damage in *mdx* mice consists of active myofiber necrosis plus the areas of subsequent regeneration (new myofibers). Values shown are average percentage (%) of whole cross-sectional area +/− SEM (values were analyzed by a *t*-test, n = 5, ^a^*P* < 0.05) [38]. ^b^*P* < 0.01.

### Fibrosis in dystrophic skeletal muscle is reduced by andrographolide treatment

**Figure 2:**
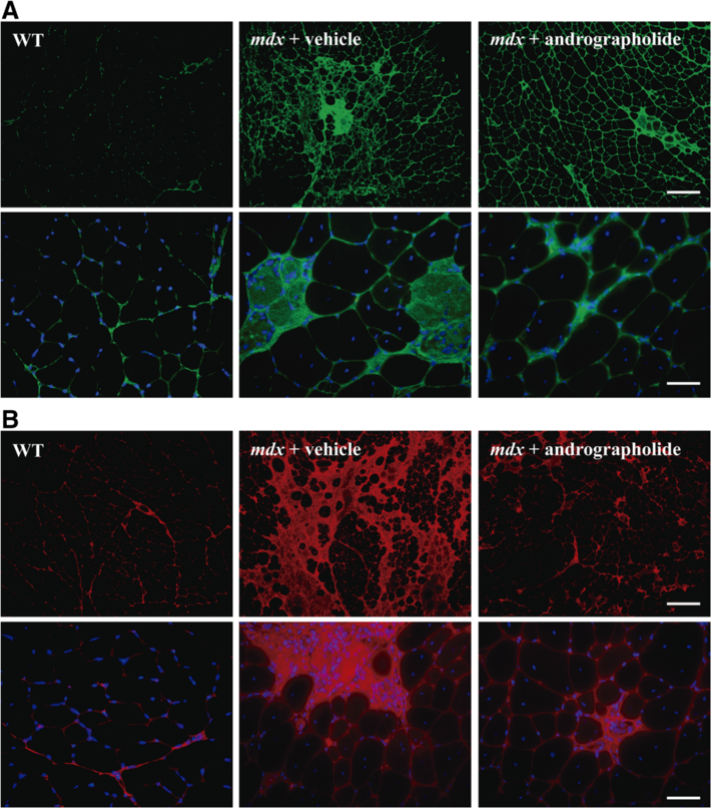
**Andrographolide reduces skeletal muscle fibrosis in *mdx *mice.** Fibrosis was augmented as explained in the legend of Figure 1. During this period, mice were treated with 1 mg/kg andrographolide or vehicle (intraperitoneal (ip) injections three times per week, six animals per group). Indirect immunofluorescence analysis of **(A)** collagen I (green) and **(B)** fibronectin (red) in cryosections of tibialis anterior (TA) muscles from wild type (WT), vehicle-treated *mdx* mice, and andrographolide-treated *mdx* mice. Nuclei are stained in blue (Hoechst). Bar corresponds to 200 and 50 μm for 100X and 400X magnification pictures respectively.

**Figure 3:**
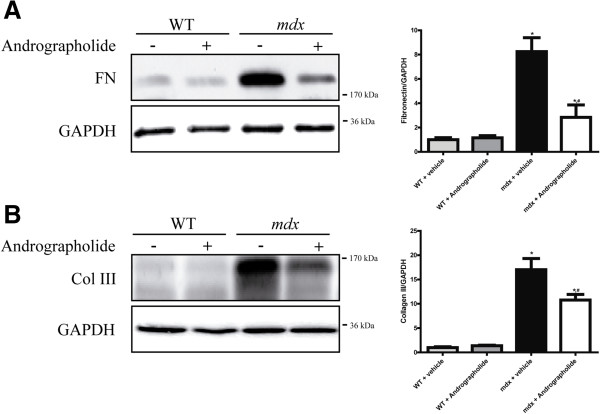
**Andrographolide reduces the amount of fibrotic proteins in *mdx *skeletal muscle.** Experiments were performed as explained in the legend of Figure 2. Muscle extracts were obtained under each experimental condition and fibrotic proteins were analyzed by western blotting. **(A)** Fibronectin (FN) and **(B)** collagen type III (Col III) from tibialis anterior (TA) muscles of wild type (WT), vehicle-treated *mdx* mice, and andrographolide-treated *mdx* mice. GAPDH protein levels are shown as loading control; molecular weight markers are shown in kDa. Values are expressed as mean ± SD of three independent experiments, using three mice for each experimental condition. (*P < 0.05 relative to WT mice; #*P* < 0.05 relative to vehicle-treated *mdx* mice).

Development of fibrosis in dystrophic skeletal muscle is characterized by an increase in ECM compounds such as fibronectin and several types of collagen [4,6,25,39]. We evaluated the effect of andrographolide on the level of fibrotic protein in skeletal muscle of dystrophic *mdx* mice. Immunofluorescence staining of TA muscles from *mdx* mice treated with andrographolide revealed a large decrease in the accumulation of collagen type III (Figure 2, upper panels) and fibronectin (Figure 2, lower panels). Western blot analysis of the same muscles indicated that andrographolide treatment decreased fibronectin and collagen III protein levels (Figure 3A and B). We also evaluated andrographolide treatment decrease in fibrosis in gastrocnemius of exercised *mdx* mice and in diaphragm from non-exercised animals, since diaphragm is the most damaged muscle in *mdx* mice and is not clearly affected by exercise [6,25]. Additional file 2: Figure S2 shows a decrease in fibrosis and damage determined by collagen type I staining and H&E respectively. Together, these results suggest that the treatment of *mdx* mice with andrographolide decreases the accumulation of fibrotic proteins in different skeletal muscle groups. Together, these results suggest that the treatment of *mdx* mice with andrographolide decreases the accumulation of fibrotic proteins.

### Andrographolide reduces the activity of NF-κB *in vivo*

**Figure 4:**
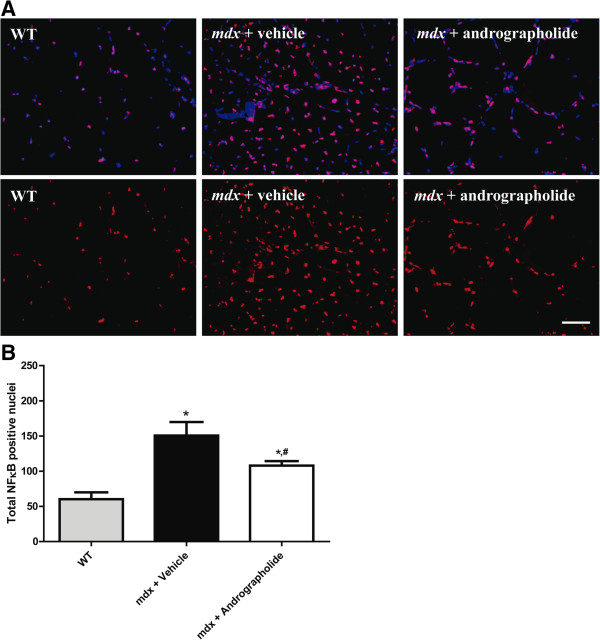
**Andrographolide decreases NF-κB activity *in vivo*.** Fibrosis was augmented as explained in the legend of Figure 1. During this period, mice were treated with 1 mg/kg andrographolide or vehicle (intraperitoneal (ip) injections three times per week, six animals per group). The activity of NF-κB was evaluated in formalin-fixed muscle samples by Southwestern blot analysis. A specific DNA was used to detect active NF-κB by immunofluorescence (red). The nuclei were stained with Hoechst (blue). **(A)** The upper panel shows merged red (active NF-κB) and blue (total nuclei) signals. Bar corresponds to 50 μm. **(B)** Quantification of total nuclei per field (in 400X magnification pictures).

Because andrographolide is a specific NF-κB inhibitor [17], we evaluated the *in vivo* activity of NF-κB in skeletal muscle of andrographolide-treated *mdx* mice by Southwestern blotting [30]. The results showed that NF-κB activity was increased in *mdx* TA compared with the corresponding wild type (WT) samples. In contrast, treatment with andrographolide reduced the number of nuclei positive for NF-κB in *mdx* TA skeletal muscle (Figure 4A and B). This result shows that andrographolide reduces the activity of NF-κB in skeletal muscle *in vivo*.

### Andrographolide reduces the level of the pro-fibrotic factor TGF-β and the downstream Smad-dependent signaling pathway in *mdx* skeletal muscles

**Figure 5:**
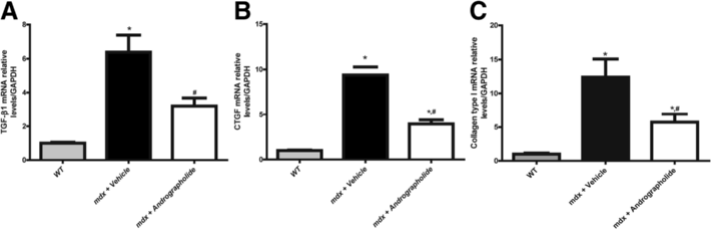
**Andrographolide reduces expression of pro-fibrotic factors and collagen type I in *mdx *mice.** Fibrosis was augmented as explained in the legend of Figure 1. During this period, mice were treated with 1 mg/kg andrographolide or vehicle (intraperitoneal (ip) injections three times per week, four animals per group). **(A)** Transforming growth factor type beta (TGF-β1), **(B)** Connective tissue growth factor (CTGF) and **(C)** collagen type I mRNA levels were determined in tibialis anterior (TA) muscle from wild type (WT), vehicle-treated *mdx* mice, and andrographolide-treated *mdx* mice by qPCR using GAPDH as a reference gene. Values correspond to the mean dCT value ± SD of three independent experiments, normalized to WT levels (*P < 0.05 relative to WT mice; #*P* < 0.05 relative to vehicle-treated *mdx* mice). The recovery scores for TGF-β1 and CTGF were 56.3% and 63.2 respectively.

TGF-β is an important pro-fibrotic factor and the downstream Smad-dependent signaling pathway is augmented in *mdx* skeletal muscle [6,40,41]. Therefore, we evaluated whether andrographolide modulated the *in vivo* expression of TGF-β. Concordant with the decrease in fibrosis, TGF-β expression determined by qPCR was reduced in *mdx* mice as a consequence of andrographolide treatment (Figure 5A). We then evaluated the expression levels of CTGF and collagen type I, two downstream pro-fibrotic factor [6,26,42] and showed that expression levels of CTGF and collagen type I in dystrophic skeletal muscle were also reduced by andrographolide treatment (Figure 5B and C).

**Figure 6:**
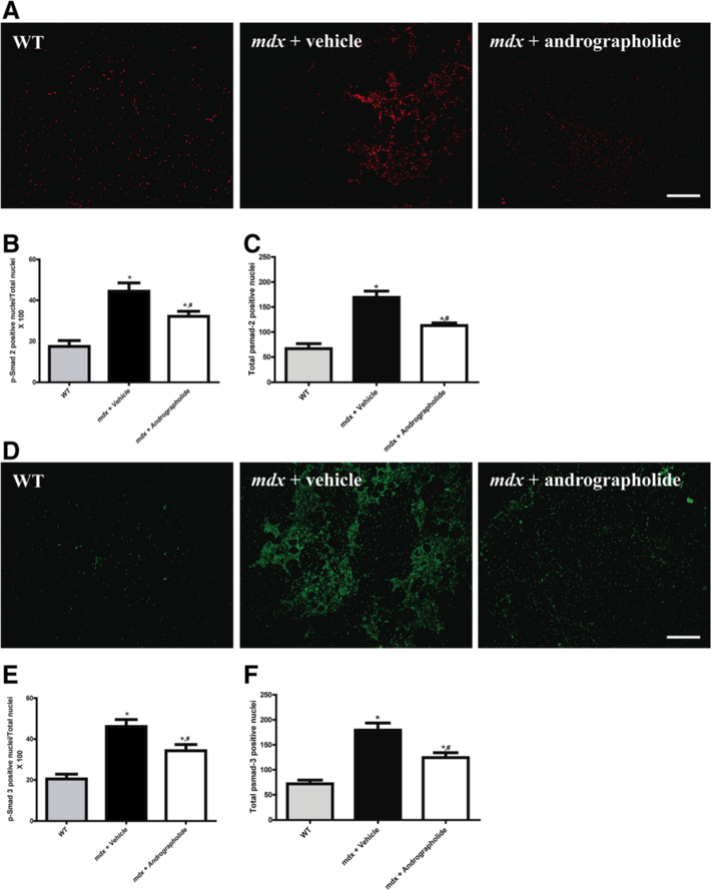
**Andrographolide reduces transforming growth factor type beta (TGF-β) signaling pathway activity in *mdx *mice.** Indirect immunofluorescence analysis of **(A)** p-Smad-2 and **(D)** p-Smad-3 (intracellular TGF-β1 mediators) in cryosections of tibialis anterior (TA) muscle from vehicle-treated and andrographolide-treated *mdx* mice. Bar corresponds to 200 μm. **(B,E)** Ratio of p-Smad-2 and p-Smad-3 respectively versus total nuclei. **(C,F)** Quantification of total nuclei per field (in 400X magnification pictures). Values are expressed as mean ± SD of three independent experiments, using three mice for each experimental condition.

We then determined whether the decrease in TGF-β expression following andrographolide treatment was reflected in diminished activity of the Smad-dependent signaling pathway. Immunofluorescence localization of phosphorylated Smad-2 and Smad-3 proteins (p-Smad-2 and p-Smad-3) in TA muscle sections indicated that andrographolide treatment reduced the number and the proportion of nuclei positive for both phosphorylated Smad proteins in TA of *mdx* mice (Figure 6). Additional file 3: Figure S3 shows the localization of p-Smad-3 in consecutive Sections. p-Smad-3 positive reaction is found mainly in necrotic-regenerating areas, likely corresponding to interstitial cells but also in nuclei inside regenerated fibers. Andrographolide treatment suggests a higher reduction in interstitial cells compared to regenerating muscle fibers. Collectively, these results show that andrographolide treatment reduced both the expression and activity of TGF-β in *mdx* skeletal muscle.

### Andrographolide treatment improves skeletal muscle strength and exercise performance in *mdx* mice

**Figure 7:**
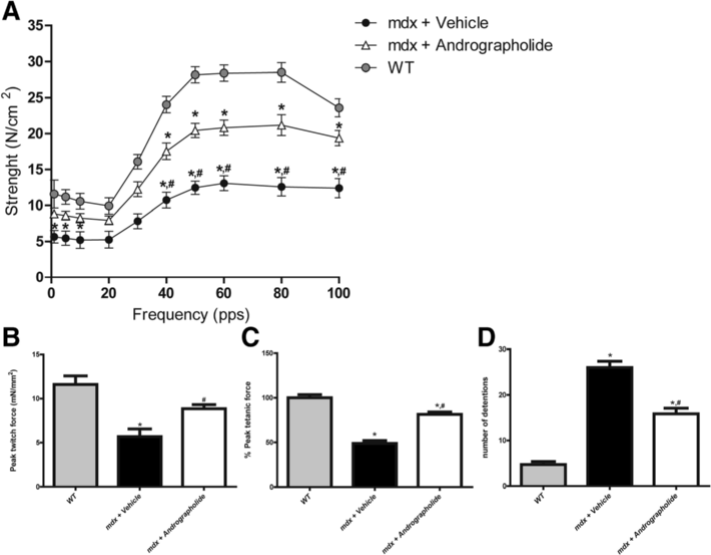
**Andrographolide increases skeletal muscle strength in *mdx *mice.** Fibrosis was augmented as explained in the legend of Figure 1. During this period, mice were treated with 1 mg/kg andrographolide or vehicle (intraperitoneal (ip) injections three times per week, six animals per group). **(A)** Tibialis anterior (TA) muscles were isolated from wild type (WT), vehicle-treated *mdx* mice, and andrographolide-treated *mdx* mice to evaluate isometric specific force (mN/mm2) at different stimulation frequencies (pulses per second, pps). **(B)** Bar graph shows tetanic specific force. Values are represented as percentage of specific isometric force generated by WT muscle (*P < 0.05 relative to WT mice; #*P* < 0.05 relative to vehicle-treated *mdx* mice). The recovery score for the twitch force measurement was 54.2%. **(C)** Bar graph showing twitch force (*P < 0.05 relative to WT mice; #*P* < 0.05 relative to vehicle-treated *mdx* mice). The recovery score for the tetanus measurement was 50.3%. **(D)** Mice were subjected to an exercise challenge on the treadmill at 15 meters/minute for five minutes and the number of set-backs was counted (n = 7, *P < 0.05 relative to WT mice; #*P* < 0.05 relative to vehicle-treated *mdx* mice). The recovery score for this measurement was 45.5%.

We then evaluated whether andrographolide treatment of *mdx* mice had an impact on the skeletal muscle physiology that determines contractile strength in isolated TA muscles. Andrographolide-treated *mdx* mice showed a significant increase in the generation of isometric force compared with vehicle-treated *mdx* mice at frequencies ranging between 50 and 100 Hz (Figure 7A). Twitch and tetanic force also showed a higher strength in the TA muscle from andrographolide-treated *mdx* mice (Figure 7B and C respectively). Given that andrographolide treatment improves muscle strength in single isolated dystrophic muscles, we asked whether andrographolide treatment similarly affects the whole body muscle performance when *mdx* mice are challenged in a treadmill running protocol. To address this question, we performed a functional test of exercise endurance through continuous exercise [23-25]. Mice treated with andrographolide showed an enhanced performance, as determined by a significant decrease in the number of detentions in the treadmill running protocol (Figure 7D). Together, these results indicate that andrographolide improves skeletal muscle strength and endurance.

### Reduction of fibrosis by andrographolide improves the efficiency of *in vivo* cell therapy

**Figure 8:**
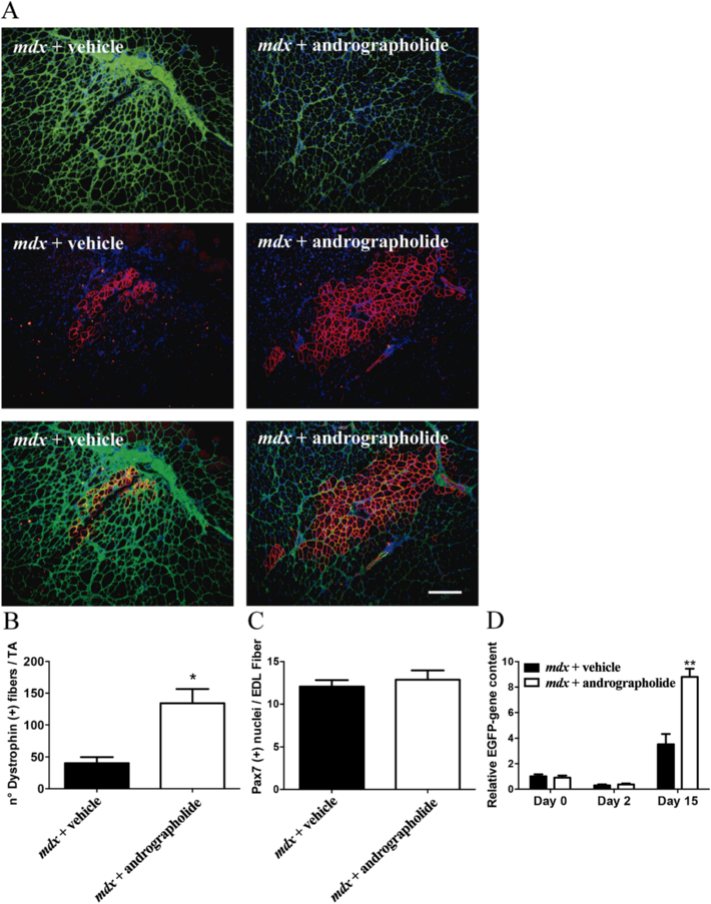
**Muscle stem cell therapy with satellite cells (SC) is improved by the reduction in muscle fibrosis following treatment with andrographolide. (A)** Fibrosis was augmented as explained in the legend of Figure 1. During this period, mice were treated with andrographolide or vehicle (intraperitoneal (ip) injections three times per week, six animals per group). One week after the last drug administration (seven-month-old mice), 500 freshly isolated satellite cells purified from wild type (WT) mice were transplanted into both tibialis anterior (TA) muscles of each *mdx* mouse. At four weeks after engraftment, the number of fibers expressing dystrophin and collagen I was determined by immunofluorescence analysis of cryosections. The images are representative of two experimental groups with six mice per group. Bar corresponds to 200 μm. **(B)** Quantification of the data obtained in (A) showing the number of myofibers expressing dystrophin per TA muscle in each case. **(C)** The number of satellite cells (PAX-7-positive nuclei) was determined for isolated single muscle fibers from extensor digitorum longus muscle (EDL) of each case as an indicator of endogenous SC survival. **(D)** 500 WT SC freshly isolated as in (A), from the C57-EGFP mice were engrafted in mice treated as in (A) (six animals per group). Both TA muscles were dissected immediately after engraftment or after 2 or 15 days from two animals in each case. Total genomic DNA was purified. EGFP transgene present in the engrafted muscle was detected by qPCR. Values are expressed as mean ± SD of three independent experiments. (*P<0.05 relative to vehicle-treated mice, **P<0.01 relative to vehicle-treated mice at day 15).

Because we observed an accumulation of fibroblasts in non-fibrotic areas of skeletal muscle from andrographolide-treated *mdx* mice, we evaluated whether cell therapy using muscle precursor cells was improved in the andrographolide-treated mice. Freshly purified satellite cells from isolated single myofibers from WT mouse donors were grafted into both TA muscles of seven-month-old *mdx* mice following pre-treatment with either andrographolide or vehicle for a three-month period under an exercise protocol. Treatment with andrographolide was stopped one week before the transplantation to rule out any direct effect of the drug on the transplanted cells. One month after the satellite cell transplantation, the muscle was analyzed for the presence of dystrophin-positive myofibers and the amount of collagen I. Figure 8 shows that the number of dystrophin-positive fibers in the *mdx* background was increased three-fold in the andrographolide-treated mice compared with controls (Figure 8A and B). Moreover, Figure 8A shows the expected reduction in collagen I content following andrographolide treatment. The purity of the transplanted cells was determined prior to the graft by plating an aliquot of the cells on ECM gel for 12 hours and analyzing expression of the muscle-specific transcription factors Pax7, MyoD, and myogenin. Over 92% of the nuclei were positive for at least one of these factors, indicating the purity of the preparation (data not shown). To evaluate if andrographolide treatment has an effect on the size of endogenous satellite cells, the number of satellite cells present on isolated EDL myofibers obtained from the same transplanted mice was determined, with a mean of 12.5 satellite cells per EDL myofiber as shown in Figure 8C, suggesting than andrographolide treatment did not affect the size of satellite cell populations. Finally the number of cells engrafted was determined as a function of time. Figure 8D, indicates that the number of transplanted cells increase with time. This increase is dramatically augmented in muscle TA obtained from mouse pre-treated with andrographolide, suggesting a higher rate of survival of the grafted cells. These results strongly suggest that andrographolide treatment of *mdx* mice improves the efficiency of cell therapy.

## Discussion

In this paper, we show that andrographolide treatment reduced skeletal muscle damage and fibrosis in *mdx* mice. We observed that this reduction was associated with an increase in muscle functionality. Moreover, we showed that the improved skeletal muscle phenotype of dystrophic mice favored the incorporation of dystrophin-positive muscle cells after intramuscular injection of satellite cells derived from WT skeletal muscle. To the best of our knowledge, this is the first report of the use of andrographolide on a model of muscular dystrophy.

Several anti-fibrotics have been tested to decrease fibrosis associated to dystrophic skeletal muscle [43]. Among them neutralizing antibodies against all three forms of TGF-β importantly reduced hydroxyproline levels and plasma creatine kinase, improved respiratory function and grip strength [44]. Halofunginone has been tested in *mdx* mice, reducing collagen content and improving respiratory and heart function. It has been suggested that it inhibits p-Smad-3 in response to TGF-β1 [45-47]. The use of inhibitors and antagonists of the renin-angiotensin system have been shown to decrease fibrosis and improve skeletal muscle function [48]. Infusion of angiotensin 1-7, which signals through the Mas receptor, has been shown to importantly decrease fibrosis, TGF-β mediated signaling and increase skeletal muscle strength [49]. It is difficult to compare which of these drugs, including andrographolide, have a better effect on dystrophic skeletal muscle, since some of them may also have other undesired side effects. Furthermore, the same readouts are not always determined in each case. Nevertheless a comparative study, under the same experimental conditions would be very valuable.

Corticosteroids are currently the most effective treatment for DMD [50-52]. They act by blocking transcription factors such as NF-κB and AP-1 to down-regulate a vast group of pro-inflammatory genes. However, the use of corticosteroids is associated with unwanted side effects and a significant proportion of patients are steroid-resistant [52], therefore there is an urgent need to develop novel anti-inflammatory drugs to replace or complement current therapy. In this study, we have preliminary data that indicate that parallel treatment with prednisone or andrographolide in *mdx* three-month-old animals for three months produce the same histological improvement together with a decrease in the amount of collagen (Additional file 4: Figure S4).

Andrographolide has been used in acute and chronic diseases such as the common cold and rheumatoid arthritis with no observable side effects (for a review, see reference [53]). Therefore, it is reasonable to consider the use of alternative treatments such as andrographolide to improve skeletal muscle physiology in patients suffering from skeletal muscular dystrophies such as DMD. Although our findings suggest that andrographolide might be used to improve quality of life in individuals with DMD, the effectiveness of andrographolide in other skeletal muscle dystrophies requires further investigation. Extrapolation of studies in small animals, such as mice, to clinical treatment of humans can be very difficult. The use of andrographolide as shown in this paper is promising because andrographolide has been used alone or as a botanical extract for the treatment of colds, fever, laryngitis and other infections with no or minimal side effects. Andrographolide not only regulates inflammation, but also suppresses the fibrotic pathology observed in chronic liver and kidney diseases [14-16]. Other advantages include the low cost of the drug, or a botanical extract highly enriched in andrographolide, and the fact that it can be given orally in capsules, thus providing hope for future therapeutic options of an oral formulation with no undesired side effects for patients with muscular disorders.

Fibrosis is one of the main features of dystrophic muscle. A high increase in ECM deposition is found in biopsies from DMD patients and in different animal models of the disease [4,39,54,55]. Although enormous efforts have been made to restore dystrophin expression in DMD patients through different approaches, such as gene and cell therapies, it has been proposed that fibrosis is an important barrier to the success of these approaches [9,21,56-58]. Therefore, targeting fibrosis is an important strategy to generate an environment that facilitates cell migration and regeneration in the dystrophic muscle. This is supported by a report that local injection of fibroblasts secreting metalloproteinases reduced collagen deposition, thereby allowing efficient subsequent therapy with intra-arterial injection of WT mesoangioblasts in dystrophic muscles [21], and the observation that models with reduced activity of a pro-fibrotic growth factor showed an increase in the number of grafted dystrophin-positive skeletal muscle fibers in *mdx* muscle [6].

Different experimental approaches have been attempted to decrease fibrosis in dystrophic muscle and other myopathies [59,60]. One of the main pro-fibrotic cytokines is TGF-β, therefore blocking TGF-β promotes histological recovery of muscle tissue and also significantly decreases the levels of ECM proteins, thus promoting and increasing muscular functionality [41,60,61]. In the same way, our results show that inhibition of fibrosis is correlated with a decrease in TGF-β. Furthermore, our results showed that andrographolide treatment inhibited the TGF-β canonical signaling pathway, as evidenced by a reduced number of nuclei positive for the key TGF-β intracellular mediators p-Smad-2 and p-Smad-3. The decrease seems to affect, in higher proportion, interstitial cells than regenerating fibers. These observations suggest a novel mechanism of the action for andrographolide.

Andrographolide is an anti-inflammatory molecule that acts through specific inhibition of NF-κB. NF-κB predominantly functions as a heterodimer of p65 and p50, in which p65 contains the transactivation domain and p50 is involved in the recognition of NF-κB DNA element responses. It has been reported that andrographolide inhibits NF-κB by covalent binding with a cysteine residue in the p50 subunit, thus inhibiting the binding of NF-κB to DNA [17]. Contrary to our report, *mdx* mice heterozygous for the p50 subunit do not present any significant histological changes; however, *mdx* mice heterozygous for the p65 subunit present a mild dystrophic phenotype [18]. A probable explanation is that p50 bound to andrographolide sequesters the p65 subunits thus preventing transactivation activity of this protein on target genes. However, this hypothesis needs to be confirmed.

Moreover, we showed that the inhibition of fibrosis by andrographolide correlates with a decrease in TGF-β and CTGF expression. Both growth factors can directly induce fibrosis in skeletal muscle and specific reduction of either of them improves the dystrophic phenotype in *mdx* mice [6,40]. It is known that TGF-β is a strong inducer of CTGF activity [7,42,62]. Furthermore, it has been shown that CTGF strongly synergizes with TGF-β to induce fibrosis [63]. CTGF expression requires NF-κB activity because the CTGF promoter contains a functional and specific NF-κB response element [64]. Therefore, is plausible that andrographolide down-regulates CTGF expression through inhibition of NF-κB because andrographolide decreased NF-κB activity *in vivo*.

## Conclusions

In conclusion, our preclinical evaluation of andrographolide in a mouse model of DMD showed promising improvements in dystrophic skeletal muscles by preventing damage and fibrosis progression. The reduction of fibrosis was associated with enhanced muscle strength and increased efficiency of cell therapy.

## Abbreviations

ANOVA: one-way analysis of variance; BSA: bovine serum albumin; CK: creatine kinase; Col: collagen; CSA: cross-sectional area; CTGF/CCN2: connective tissue growth factor; DIG: digoxigenin; DMD: Duchenne Muscular Dystrophy; DMEM: Dulbecco’s modified Eagle’s medium; EBD: Evans blue dye; ECM: extracellular matrix; EDL: extensor digitorum longus; FN: fibronectin; H&E: hematoxylin-eosin staining; ip: intraperitoneal; NF-κB: nuclear factor kappa-light-chain-enhancer of activated B cells; PBS: phosphate-buffered saline; qPCR: quantitative real-time polymerase chain reaction; SC: satellite cells; TA: tibialis anterior; TGF-β: transforming growth factor type beta; WT: wild type.

## Competing interests

The authors declare that they have no competing interests.

## Authors’ contributions

DC carried out the andrographolide experiments in mice, participated in the sequence alignment and drafted the manuscript. JG carried out the grafting assays. CC-V participated in TGF-β analysis. MGM participated in the CTGF analysis. SM participated in the NF-κB analysis. JCC, RF and JH participated in the discussion of the design of the experiments. EB conceived of the study, and participated in its design and coordination and helped to draft the manuscript. All authors read and approved the final manuscript.

## Supplementary Material

Additional file 1: Figure S1 TA fiber diameter in *mdx* mice is not affected by andrographolide treatment. Minimal Feret’s diameters were determined in muscle cross- sections from WT, *mdx* mice treated with vehicle, and *mdx* mice treated with andrographolide. Fiber diameters were grouped from 0 to 100 μm. The images are representative of three independent experiments, using four mice for each experimental condition. Click here for file

Additional file 2: Figure S2 Andrographolide improves skeletal muscle morphology and reduces the amount of collagen I in *mdx* skeletal muscles. (A) Fibrosis was augmented as explained in the legend of Figure 1. During this period, mice were treated with 1 mg/kg andrographolide or vehicle (ip injections three times per week, six animals per group). H&E staining and indirect immunofluorescence analysis of collagen I (green) in cryosections of gastrocnemius muscles from vehicle-treated *mdx* mice and andrographolide-treated *mdx* mice are shown in the upper and the bottom panel respectively. (B) Non-exercised mice were treated with 1 mg/kg andrographolide or vehicle (ip injections three times per week, six animals per group). H&E staining and indirect immunofluorescence analysis of collagen I (green) in cryosections of diaphragm muscles from vehicle-treated *mdx* mice and andrographolide-treated *mdx* mice are shown in the upper and the bottom panel respectively. Bar corresponds to 50 μm. Nuclei are stained in blue (Hoechst). Click here for file

Additional file 3: Figure S3 Andrographolide mainly reduces TGF-β signaling pathway activity in necrotic and regeneration foci in *mdx*. Upper panel shows an indirect immunofluorescence analysis of p-Smad-3 (red), to localize the positive nuclei, the membranes were labeled with wheat germ agglutinin (green) and the nuclei are stained in blue (Hoechst). Bottom panel shows consecutive sections stained with H&E. Bar corresponds to 200 μm. Click here for file

Additional file 4: Figure S4 Andrographolide or prednisone treatment improves skeletal muscle histology and decreases collagen content to some extent in *mdx* skeletal muscles. Fibrosis was augmented as explained in the legend of Figure 1. During this period, mice were treated with 1 mg/kg andrographolide or with 5 mg/kg prednisone (both drugs were administered orally twice weekly on consecutive days). The figure shows an indirect immunofluorescence analysis of collagen I (green) in cryosections of TA muscles from vehicle-treated *mdx* mice, andrographolide-treated *mdx* mice and prednisone-treated *mdx* mice. Nuclei are stained in blue (Hoechst). Bar corresponds to 50 μm. Click here for file
