# Supplementary figures and images for: Andrographolide attenuates skeletal muscle dystrophy in mdx mice and increases efficiency of cell therapy by reducing fibrosis
Source: Skelet Muscle. 2014 Mar 21;4:6. doi: 10.1186/2044-5040-4-6 (PMC4021597; doi:10.1186/2044-5040-4-6)

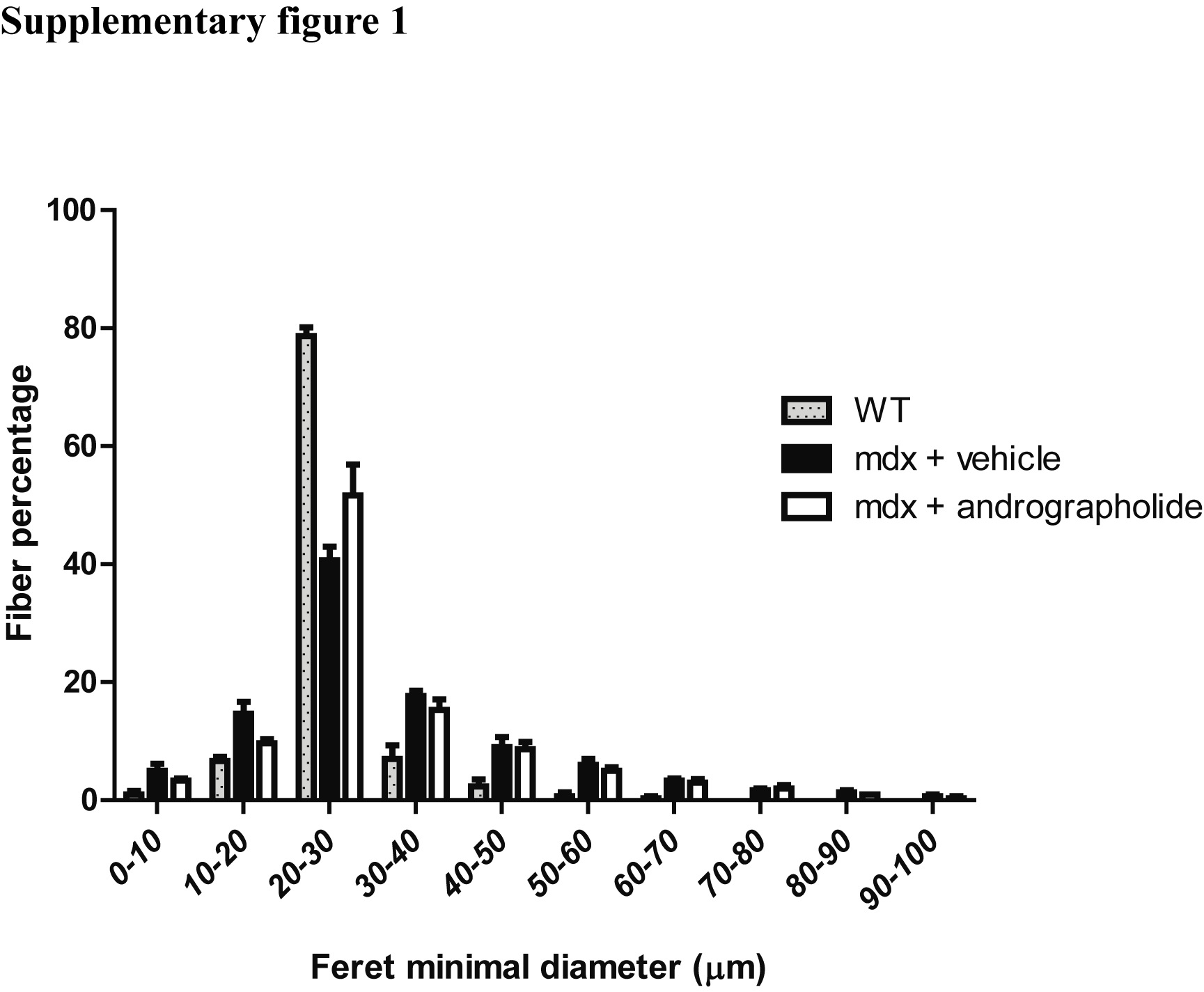

Supplement: Additional file 1: Figure S1 — TA fiber diameter in mdx mice is not affected by andrographolide treatment. Minimal Feret’s diameters were determined in muscle cross- sections from WT, mdx mice treated with vehicle, and mdx mice treated with andrographolide. Fiber diameters were grouped from 0 to 100 μm. The images are representative of three independent experiments, using four mice for each experimental condition. [file 2044-5040-4-6-S1.tiff]

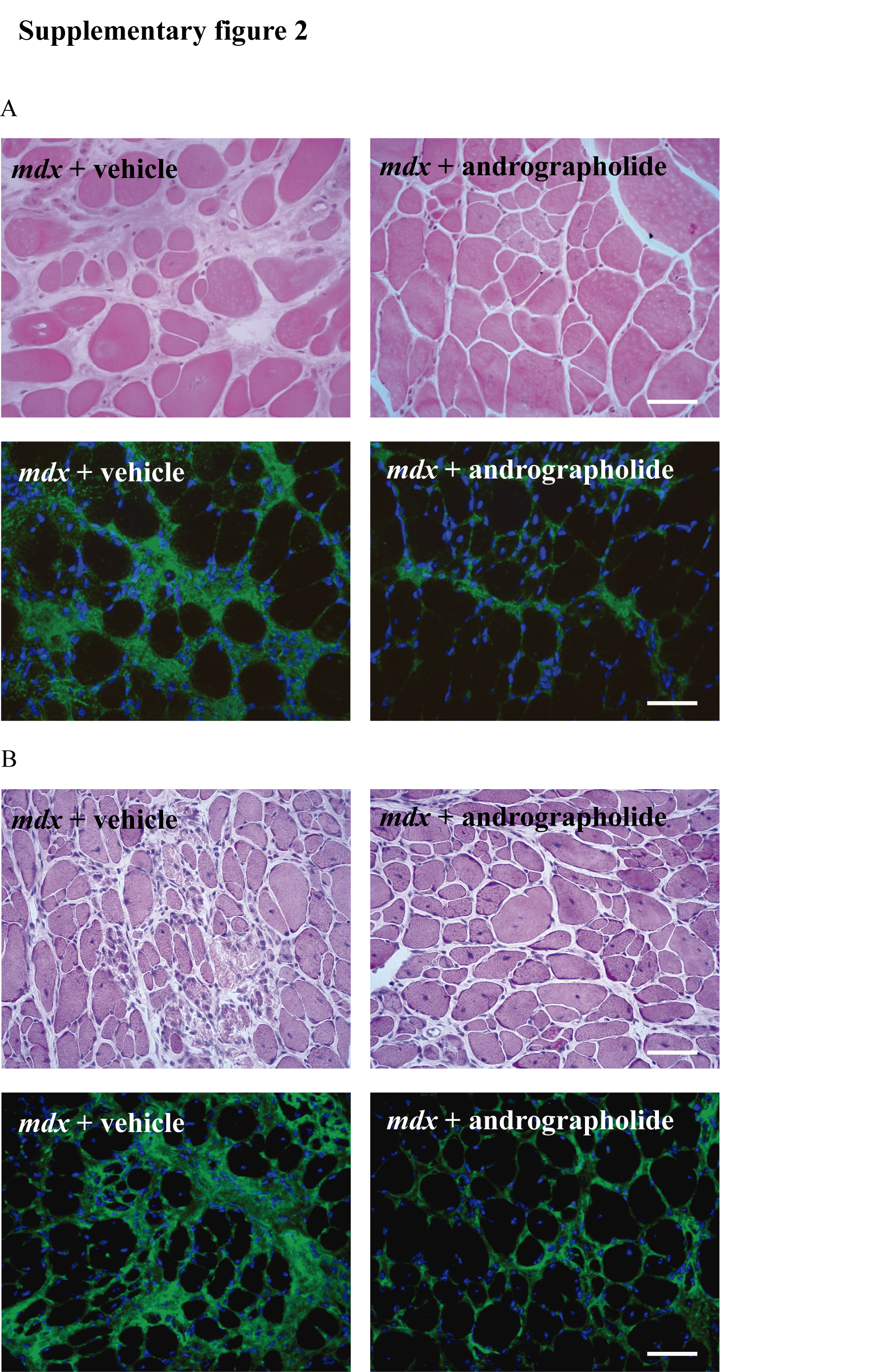

Supplement: Additional file 2: Figure S2 — Andrographolide improves skeletal muscle morphology and reduces the amount of collagen I in mdx skeletal muscles. (A) Fibrosis was augmented as explained in the legend of Figure 1. During this period, mice were treated with 1 mg/kg andrographolide or vehicle (ip injections three times per week, six animals per group). H&E staining and indirect immunofluorescence analysis of collagen I (green) in cryosections of gastrocnemius muscles from vehicle-treated mdx mice and andrographolide-treated mdx mice are shown in the upper and the bottom panel respectively. (B) Non-exercised mice were treated with 1 mg/kg andrographolide or vehicle (ip injections three times per week, six animals per group). H&E staining and indirect immunofluorescence analysis of collagen I (green) in cryosections of diaphragm muscles from vehicle-treated mdx mice and andrographolide-treated mdx mice are shown in the upper and the bottom panel respectively. Bar corresponds to 50 μm. Nuclei are stained in blue (Hoechst). [file 2044-5040-4-6-S2.tiff]

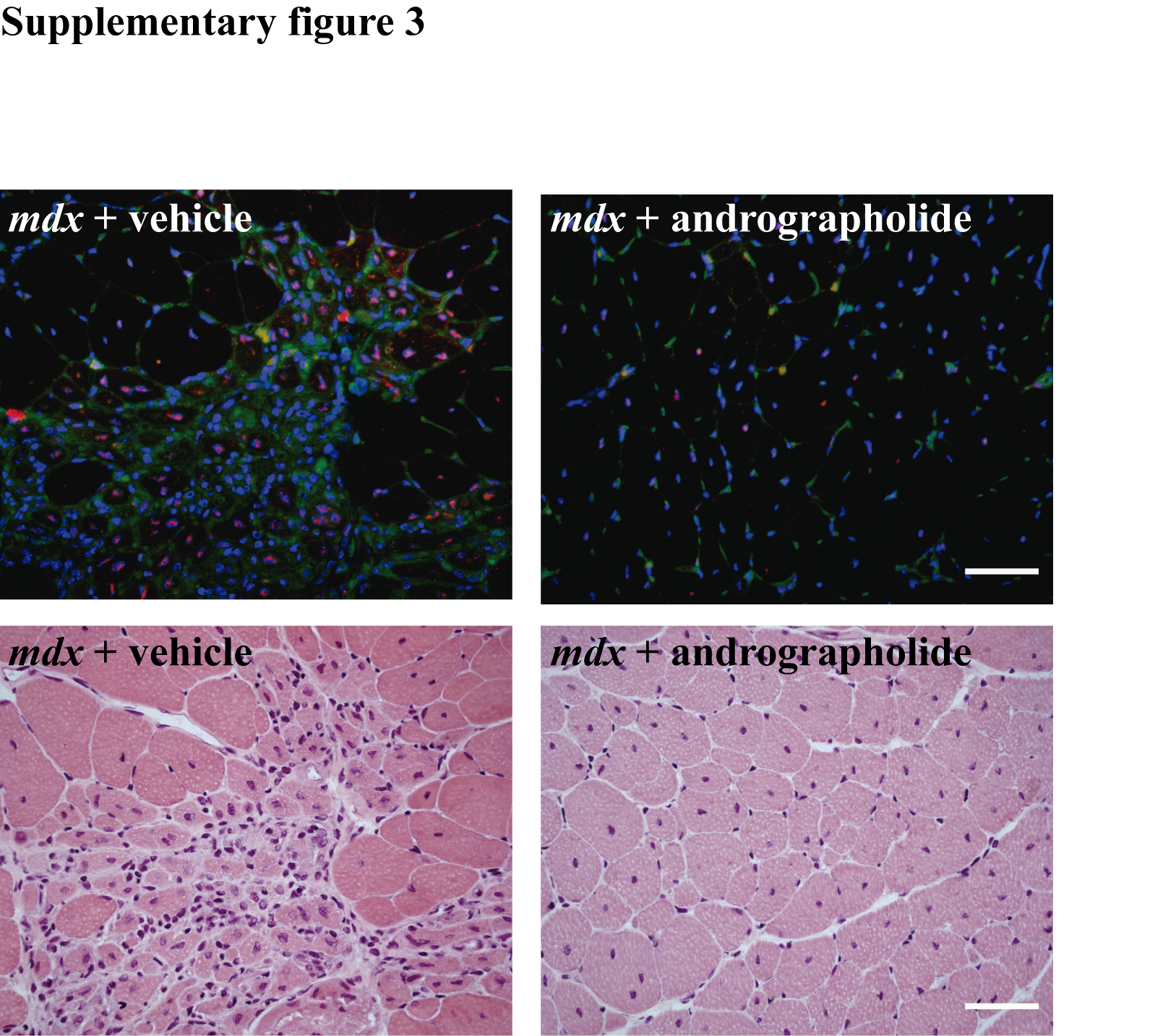

Supplement: Additional file 3: Figure S3 — Andrographolide mainly reduces TGF-β signaling pathway activity in necrotic and regeneration foci in mdx. Upper panel shows an indirect immunofluorescence analysis of p-Smad-3 (red), to localize the positive nuclei, the membranes were labeled with wheat germ agglutinin (green) and the nuclei are stained in blue (Hoechst). Bottom panel shows consecutive sections stained with H&E. Bar corresponds to 200 μm. [file 2044-5040-4-6-S3.tiff]

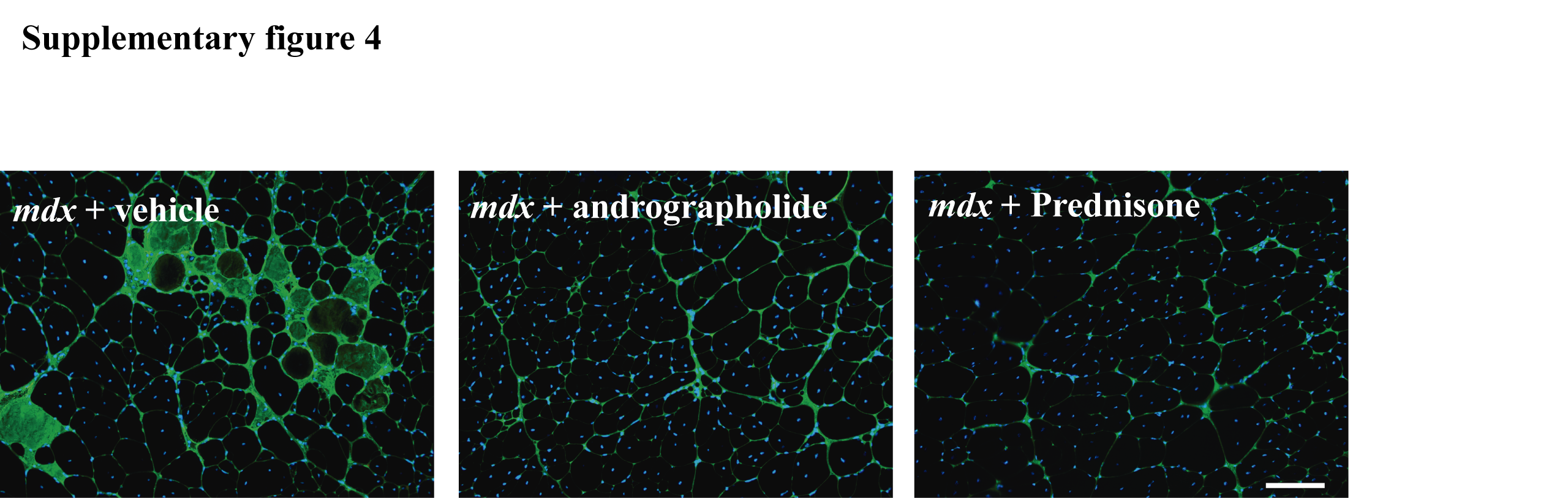

Supplement: Additional file 4: Figure S4 — Andrographolide or prednisone treatment improves skeletal muscle histology and decreases collagen content to some extent in mdx skeletal muscles. Fibrosis was augmented as explained in the legend of Figure 1. During this period, mice were treated with 1 mg/kg andrographolide or with 5 mg/kg prednisone (both drugs were administered orally twice weekly on consecutive days). The figure shows an indirect immunofluorescence analysis of collagen I (green) in cryosections of TA muscles from vehicle-treated mdx mice, andrographolide-treated mdx mice and prednisone-treated mdx mice. Nuclei are stained in blue (Hoechst). Bar corresponds to 50 μm. [file 2044-5040-4-6-S4.tiff]
